# P04.51. Study of natural health product adverse reactions (SONAR): active surveillance in community pharmacies

**DOI:** 10.1186/1472-6882-12-S1-P321

**Published:** 2012-06-12

**Authors:** C Necyk, H Boon, B Foster, W Jaeger, D LeGatt, G Cembrowski, M Murty, D Vu, R Leitch, R Tsuyuki, J Barnes, T Charrois, J Arnason, M Ware, R Rosychuk, S Vohra

**Affiliations:** 1 University of Alberta, Edmonton, Canada; 2 Leslie Dan Faculty of Pharmacy, University of Toronto, Toronto, Canada; 3 Faculty of Medicine, University of Ottawa, Ottawa, Canada; 4 Department of Medicine, University of Alberta, Edmonton, Canada; 5 Department of Laboratory Medicine and Pathology, University of Alberta, Edmonton, Canada; 6 Health Canada, Ottawa, Canada; 7 Department of Medicine, University of Alberta, Edmonton, Canada; 8 School of Pharmacy, University of Auckland, Auckland, New Zealand; 9 School of Pharmacy, Curtin University of Technology, Perth, Australia; 10 Department of Biology, University of Ottawa, Ottawa, Canada; 11 Research Institute, McGill University Health Centre, Montreal, Canada; 12 Department of Pediatrics, University of Alberta, Edmonton, Canada

## Purpose

To investigate the adverse event (AE) rates associated with natural health product (NHP) use, prescription drug use and concurrent NHPs-drug use through active surveillance in community pharmacies in Alberta and British Columbia, Canada.

## Methods

Participating pharmacists and pharmacy technicians screened consecutive individuals picking up prescription medications about their (1) NHP use, (2) prescription medication use, (3) concurrent NHP/prescription medication use in the previous one month, and (4) the occurrence of potential AEs. If a potential AE was identified and the patient provided written consent, a research pharmacist conducted a guided telephone interview to gather additional detailed information on the AE and medical history of the patient.

## Results

Over a total of 105 pharmacy weeks, 1119 patients were screened. Of these patients, 409 reported taking prescription drugs only (36%; 95% CI: 33.7-39.4), 41 reported taking NHPs only (3.7%; 95% CI: 2.6-4.8) and 656 reported taking NHPs and prescription medication concurrently (58.6%; 95% CI: 55.7 to 61.5). A total of 58 patients reported a possible AE, which represents 0.98% (95% CI: 0.03 to 1.93) of those taking prescription medications only, 9.8% of those taking NHPs only (95% CI: 0.7% to 18.9) and 7.5% of those taking NHPs and prescription medications concurrently (95% CI: 5.48 to 9.52).

## Conclusion

Compared to passive surveillance, this study found active surveillance to markedly improve NHP adverse event reporting rates. Active surveillance offers improved quantity and quality of adverse event data, allowing for meaningful adjudication to assess potential harms.

